# Global, Regional, and National Burden and Trends of Down Syndrome From 1990 to 2019

**DOI:** 10.3389/fgene.2022.908482

**Published:** 2022-07-15

**Authors:** Liyuan Chen, Lifei Wang, Yi Wang, Haishan Hu, Yuan Zhan, Zhilin Zeng, Lidan Liu

**Affiliations:** ^1^ Department of Obstetrics and Gynecology, Wuhan No 1 Hospital, Wuhan, China; ^2^ Second Clinical College, Tongji Hospital, Tongji Medical College, Huazhong University of Science and Technology, Wuhan, China; ^3^ Department of Reproductive Medicine, The First Affiliated Hospital of Hainan Medical University, Hainan Medical University, Wuhan, China; ^4^ Department of Respiratory and Critical Care Medicine, National Clinical Research Center of Respiratory Disease, Tongji Hospital, Tongji Medical College, Huazhong University of Science and Technology, Wuhan, China; ^5^ Department and Institute of Infectious Diseases, Tongji Hospital, Tongji Medical College, Huazhong University of Science and Technology, Wuhan, China

**Keywords:** down syndrome, GBD 2019, social demographic index, incidence, mortality, disability-adjusted life years

## Abstract

**Introduction:** Down syndrome (DS) is the leading cause of genetically defined intellectual disability and congenital birth defects worldwide. A large population of people diagnosed with DS globally is posing an enormous socioeconomic burden. However, the global burden and trends of DS have not been reported.

**Methods:** Based on the data from the Global Burden of Disease database in 2019, we analyzed the incidence, prevalence, disability-adjusted life years (DALYs), and death of DS from 1990 to 2019 according to sex, age, regions, and social-demographic index (SDI). Then, age-standardized rates (ASRs) and estimated annual percentage change (EAPC) of these aforementioned indexes were calculated to evaluate the temporal trend of DS. Finally, the association of SDI with DS epidemiological parameters was assessed.

**Results:** In the past 30 years, the incident cases, age-standardized incident rate (ASIR), and age-standardized prevalent rate (ASPR) of DS first decreased slightly and subsequently increased globally. The number of prevalent cases increased steadily, while the number and age-standardized rate (ASRs) of DALYs and deaths decreased gradually from 1990 to 2019. In the meantime, disease burdens were different across various SDI regions. The prevalent cases and ASPR for both sexes were increasing in all SDI regions except for the high-middle SDI region. At the national level, Brunei Darussalam, Ireland, and Haiti were the top three countries with the highest ASIR in 2019. Georgia was in the top three with the highest increase in ASRs of four parameters, while Serbia was consistently ranked in the top three with fastest declining. Furthermore, we found that ASIR and ASPR were positively correlated with SDI, yet the age-standardized DALYs and age-standardized death rate (ASDR) were negatively correlated with SDI.

**Conclusion:** In the past 30 years, the burden and trends of DS were heterogeneous across different regions and countries with different sociodemographic characteristics. Great improvements had been achieved in reducing DALYs and deaths globally. However, the increased number and ASRs of incident and prevalent cases in some regions, especially in low SDI regions, were contributing to numerous challenges to public health. The findings may provide valuable information to the development or implementation of more effective measures.

## Introduction

Annually, an estimated 7.9 million children are born with a severe birth defect due to genetic or partially genetic origin (Christianson et al., 2006). Every year, an estimated number of a minimum of 3.3 million children under 5 years of age die from birth defects and 3.2 million survivors may be disabled for life (Christianson et al., 2006). The most common severe aneuploid condition at the time of birth is Down syndrome (DS) (Arbuzova et al., 2002), which was first described by the British physician Dr. John Langdon H. Down in 1866 (Mercer et al., 2004). The presence of extra chromosome 21 has been recognized as the cause of DS, manifested with mental and motor developmental impairment, facial dysmorphia, and congenital malformations, which is often accompanied by congenital heart disease (Verstegen and Kusters, 2020). The onset of DS usually occurs during prenatal development (Dierssen, 2012), with approximately one case in 1,000 births (Grimm et al., 2021). DS is generally diagnosed prenatally or at the time of birth by means of cell-free prenatal screening with parallel sequencing of maternal plasma cell-free DNA or genetic karyotype testing (Grieco et al., 2015; Bull, 2020). DS patients have an increased susceptibility to develop infections, autoimmune disorders, and hematologic and oncologic abnormalities (Lal et al., 2015; Verstegen and Kusters, 2020). Respiratory infection is the most common reason of death in childhood with DS (McDowell and Craven, 2011; Bull, 2020), while dementia is the direct reason of death in 70% older people with DS (Bull, 2020; McGlinchey et al., 2020). Individuals with DS are usually institutionalized; therefore, family is suffering from a heavy nursing and economic burden (Megarbane et al., 2009). Involvement in community life has become increasingly important as persons with DS survive longer and achieve greater degrees of independence (Churchill et al., 2012). An emphasis on transitions of employment, source of health care, and community involvement, as well as on legal issues and financial support, has been found to be essential for the long-term well-being of persons with DS and their families (Nugent et al., 2018). In addition, evidence-based clinical guidelines have been developed to provide recommendation to support primary care of adults with DS (Tsou et al., 2020). Owing to elevated consciousness, modified treatment protocols, and advanced social supportive medical care (Verstegen et al., 2020), the average life expectancy for persons with DS is on the rise from 25 years in 1983 to 60 years in 2020 (Tsou et al., 2020).

As the average age of pregnant women increased, the number of fetuses with DS had risen (Roizen and Patterson, 2003). The prevalence of DS is correlated positively with maternal age and inversely with gestational age (De Leon-Luis et al., 2014). It was estimated that there is an overall 30% reduction in the numbers of babies with DS from 2006 to 2010 due to elective pregnancy terminations. It is well known that the practice of prenatal screening and selective termination of pregnancy have exerted significant impact on the burden of DS. In addition, researchers also found that lower participation rates are obtained in the prenatal test among women from a lower socioeconomic background (Kuppermann et al., 2006). The percentage of women aged greater than 35, who do not have universal screening, prenatal diagnosis, and associated services, was high in middle- and low-income countries (Christianson et al., 2006). In addition, many studies indicated that sociodemographic characteristics impacted the survival and the risk of mortality for patients with DS (Fiscella et al., 2000; de Campos Gomes et al., 2020)**.** Therefore, maternal age, participation rates in prenatal screening and selective termination of pregnancy, and sociodemographic index (SDI) level were associated with burden of DS.

As one of the important public health issues worldwide, DS imposes a heavy burden on the family and society. However, there is lack of research studies to assess the global burden of DS at present, to our knowledge. In this study, we aim to show the global burden and epidemiology trend of DS stratified by age, SDI, regions, and countries from 1990 to 2019.

## Methods

### Definition

As the unit of analysis for measuring the relative magnitude of losses of healthy life associated with specific causes, the disability-adjusted life year (DALYs) is a summary measure of the years lived with disability and the years of life lost (Salomon, 2014). The DALYs has been proposed by the World Bank and the WHO as a measure of the global impact of disease on individual illness status (FerrucciC et al., 2007).

SDI, scaled from 0 to 1, is a composite indicator of overall development based on the rankings of incomes per capita, years of schooling, and fertility rates in females younger than 25 years. The larger the SDI is, the more developed the country is. Age-standardized rates (ASRs) refer to the method of statistical processing of demographic data according to the same standard age composition (Mohsen Naghavi et al., 2015). The purpose is to eliminate the influence of different age compositions of the population among different geographical areas and ensure comparability of statistical indicators (Omar et al., 2001).

### Data Acquisition

We collected the annual incident cases, prevalent cases, the DALYs and death, and the age-standardized data of DS from 1990 to 2019 from the Global Burden of Disease (GBD) 2019, which measures epidemiological levels and trends among communicable diseases, noncommunicable diseases, and injuries globally (Diseases and Injuries, 2020). The methodology of GBD 2019 study has been described in previous studies (Diseases and Injuries, 2020).

Information on age, SDI and geographic location was also obtained to further analyze the disease burden. To better exhibit the age distribution of DS burden, we divided population into 4 groups according to age, including those below 5 years old, 5–14 years old, 15–49 years old, and above 50 years old. In order to explore the association between SDI and DS burden, countries and territories were divided into five regions according to the SDI, including namely the low, low-middle, middle, high-middle and high SDI. Moreover, the world was divided into 21 regions according to the geographic location.

### Statistical Analysis

The Institutional Review Board of Wuhan No.1 Hospital determined that approval was waived because of publicly available data. The GBD 2019 complies with the Guidelines for Accurate and Transparent Health Estimates Reporting statement (Stevens et al., 2016). The standardized methods of the GBD 2019 have been extensively reported (Diseases and Injuries, 2020). Annual number of incident cases, prevalent cases, death and DALY, and corresponding ASRs (number per 100,000 population) were used to describe the disease burden. Incidence measures the rapidity of disease occurrence, while prevalence measures the proportion of the population with disease.

ASRs were calculated on the basis of the following formula: $ASRs=\frac{\sum_{\text{i}=1}^{A}a_{i}w_{i}}{\sum_{\text{i}=1}^{A}w_{i}}\text{×}100\text{,}000$ The ASRs are equal to the sum of the product of the specific age ratio (a_i_) in age group i and the number (or weight) (w_i_) of the selected reference standard population group i divided by the sum of number (or weight) of the standard population (Chen et al., 2021).

The estimated annual percentage change (EAPC) was calculated using the following regression model to assess the trends of ASRs: Y = α+βX+ε, where Y refers to ln (ASRs), X represents calendar year, ε means error term, and β determines the positive or negative trends in ASRs. The EAPC could be given by 100*(exp(β)-1). The ASRs were considered to be on the rise when the estimation of EAPC and its lower boundary of 95% uncertainty interval [UI] were both positive. On the contrary, the ASRs were considered to be in a downward trend when EAPC and its upper boundary of 95% UI were both negative. Otherwise, the ASRs were considered to be stable over time (Chen et al., 2021).

The Spearman’s correlation coefficients were used to assess the relationships between the ASRs and SDI. In the correlation analysis, if the Pearson correlation coefficient was >0 and the *p* value was <0.05, there was a significant positive correlation between the two variables. The maps were made using ECharts software. The *p*-value less than 0.05 is considered statistically significant.

## Results

### Incidence of Down Syndrome

**FIGURE 1:**
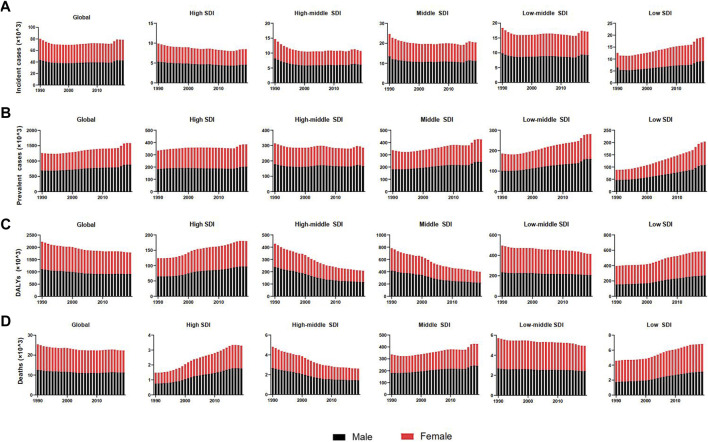
Burden and trends of Down syndrome globally and in five SDI quintiles from 1990 to 2019. **(A)** Incident cases. **(B)** Prevalent cases. **(C)** Disability-adjusted life-years (DALYs). **(D)** Deaths. Black bars represent males, and red bars represent females. Note: DALYs, disability-adjusted life-years; SDI, social-demographic index.

As presented in Figure 1A and Supplementary Figure S1A, there were almost no changes both in the incidence cases and the age-standardized incident rate (ASIR) of DS from 80,060 (95% uncertainty interval [UI] 61,960-102,450) and 1.22 (95% UI 0.94–1.56) per 100,000 population in 1990 to 78,430 (95% UI 60,130-101,730) and 1.21 (95% UI 0.93–1.57) in 2019, respectively. Although the EAPC (0.03, 95% UI -0.14-0.20) of ASIR signified the basically unchanged trend from 1990 to 2019 worldwide, both of incident cases and ASIR were first in downward trends and then in upward with the turning point in 2001 (69,638, 95% UI 54,913-87,017) and 1996 (1.08, 95% CI 0.86–1.35), respectively (Supplementary Table S1 and Supplementary Table S7).

**FIGURE 2:**
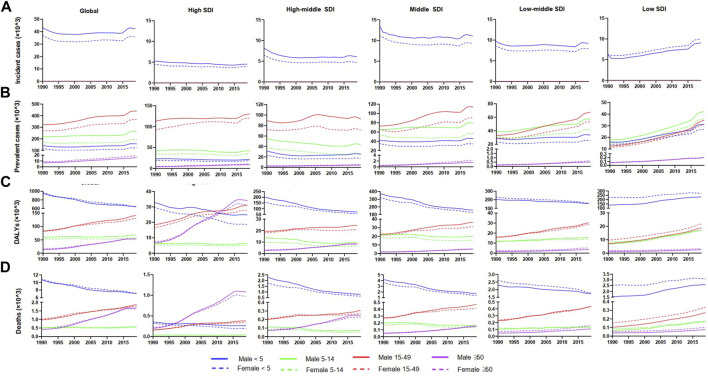
Change trends of Down syndrome incident cases, prevalent cases, DALYs, and deaths from 1990 to 2019 in different age groups. **(A)** Change trends of incident cases. **(B)** Change trends of prevalent cases. **(C)** Change trends of DALYs. **(D)** Change trends of deaths. Note: DALYs, disability-adjusted life-years.

**FIGURE 3:**
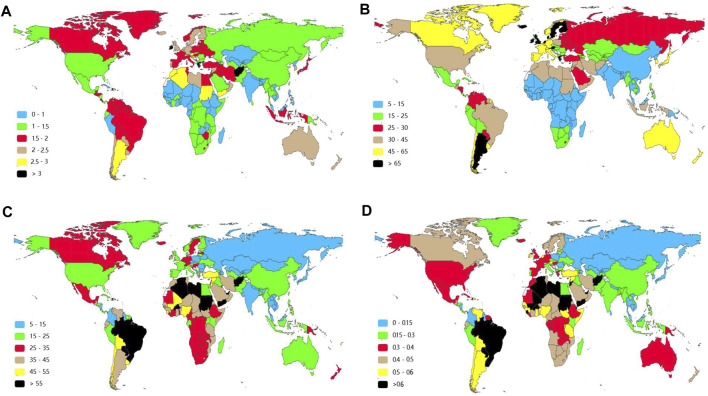
Age-standardized rate of Down syndrome in 2019 in 204 countries and territories. **(A)** Age-standardized incident rate (ASIR). **(B)**Age-standardized prevalent rate (ASPR). **(C)** Age-standardized DALY rates. **(D)** Age-standardized death rates (ASDRs).

**FIGURE 4:**
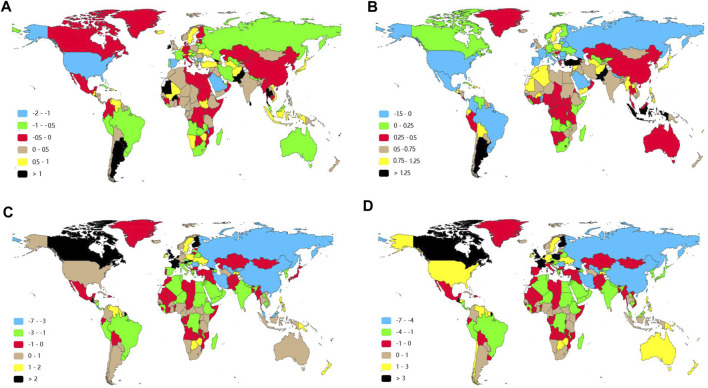
Estimated annual percentage changes of Down syndrome in 204 countries and territories between 1990 and 2019. **(A)** EAPC of age-standardized incident rates (ASIRs). **(B)** EAPC of age-standardized prevalent rates (ASPRs). **(C)** EAPC of age-standardized disability-adjusted life-year (DALY) rates. **(D)** EAPC of age-standardized death rates (ASDRs). Note: DALYs, disability-adjusted life-years; EAPC, estimated annual percentage changes.

**FIGURE 5:**
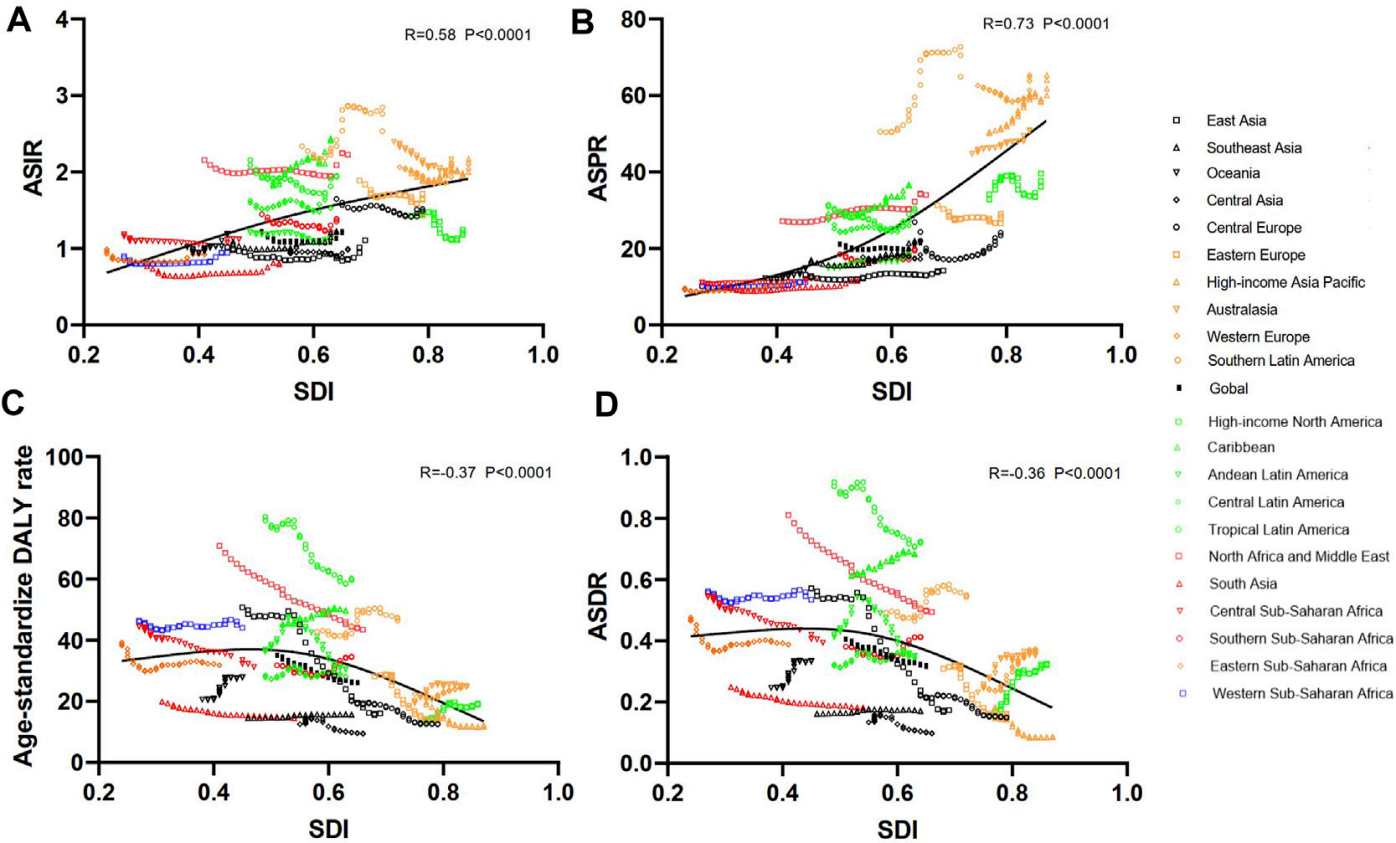
Association between ASRs of Down syndrome and SDI in 21 regions from 1990 to 2019. The SDI was positively correlated with the ASIR **(A)** and ASPR **(B)** in 21 regions from 1990 to 2019. The SDI was negatively correlated with the age-standardized DALY rate **(C)** and ASDR **(D)**. Note: ASRs, age-standardized rates; ASIR, age-standardized incident rate; ASPR, age-standardized prevalent rate; ASDR, age-standardized death; DALYs, disability-adjusted life-years; SDI, social-demographic index.

The incidences of different SDI regions were divergent while males had more incident cases and ASIR from DS than females except for the low SDI region (Figure 1A, Supplementary Figure S1A, Figure 2A and Table 1). The middle SDI region had the most incidence cases in 1990 (24,610, 95%UI 19,070-32,050) and 2019 (20,450, 95%UI 15,610-26,390). The low-middle SDI region held the lowest ASIR both in 1990 (1.01, 95%UI 0.76–1.31) and in 2019 (1.01, 95%UI 0.77–1.32). The high SDI region simultaneously obtained the lowest incident cases and the most ASIR at that time in the past 3 years (Table 1). Interestingly, the high SDI region with the most ASIR had the biggest downward trend (EAPC -0.28, 95%UI -0.40 to -0.16) from 1990 to 2019 (Table 1). Among the 21 regions divided according to geographical characteristics, the ASIR of South Asia maintained the lowest value both in 1990 (0.77, 95%UI 0.57–1.01) and in 2019 (0.79, 95%UI 0.59–1.04), but the Australasia with the highest ASIR in 1990 (2.40, 95%UI 1.90–2.96) was replaced to Southern Latin America (2.53, 95%UI 2.02–3.17) in 2019, which also had the highest growth rate with EAPC value of 1.02 (95%UI 0.74–1.30). High-income North America declined fastest in ASIR from 1990 to 2019 with EAPC value of -1.06 (95%UI −1.31 to −0.81) (Figure 3A, Figure 4A, Table 1). The top three countries with the highest ASIR in 2019 were Brunei Darussalam (3.94, 95%UI 3.02–4.97), Ireland (3.80, 95%UI 2.73–5.14), and Haiti (3.54, 95%UI 2.50–5.00), while America had the lowest ASIR (0.60, 95%UI 0.46–0.80) among all the countries (Figure 3A, Supplementary Table S3). The ASIR rose fastest in Georgia (EAPC 2.36, 95%UI 2.00–2.72) and decreased fastest in Serbia (EAPC -2.09, 95%UI -2.22 to -1.96) (Figure 4A, Supplementary Table S4). In Figure 5A, ASIR and SDI had a positive correlation (R = 0.58, *p* < 0.0001), which meant ASIR seems to be higher in relatively developed regions.

**TABLE 1:** Incidence of Down syndrome in 1990/2019 and temporal trends.

Characteristic	1990				2019			1990–2019
	Incident cases no.×10^3^ (95% CI)		ASIR/10^5^ no. (95% CI)		Incident cases no.×10^3^ (95% CI)		ASIR/10^5^ no. (95% CI)	EAPC no. (95% CI)
Overall	80.06 (61.96–102.45)		1.22 (0.94–1.56)		78.43 (60.13–101.73)		1.21 (0.93–1.57)	0.03 (-0.14–0.2)
Sex								
Male	43.15 (33.35–55.65)		1.27 (0.98–1.64)		42.57 (32.66–55.36)		1.27 (0.98–1.65)	0.04 (-0.14–0.21)
Female	36.91 (28.55–47.2)		1.16 (0.9–1.48)		35.86 (27.47–46.62)		1.15 (0.88–1.49)	0.03 (-0.14–0.2)
Socio-demographic factor								
High SDI	9.86 (7.87–12.14)		1.74 (1.39–2.14)		8.47 (6.68–10.41)		1.71 (1.35–2.1)	-0.28 (-0.4∼-0.16)
High-middle SDI	14.63 (11.46–18.69)		1.47 (1.15–1.88)		10.7 (8–13.82)		1.42 (1.06–1.83)	-0.04 (-0.18–0.1)
Middle SDI	24.61 (19.07–32.05)		1.19 (0.92–1.55)		20.45 (15.61–26.39)		1.19 (0.91–1.54)	0.11 (-0.05–0.26)
Low-middle SDI	18.36 (13.74–23.86)		1.01 (0.76–1.31)		17.14 (13–22.41)		1.01 (0.77–1.32)	0.19 (-0.02–0.39)
Low SDI	12.53 (9.12–16.51)		1.11 (0.81–1.46)		19.08 (13.89–25.72)		1.06 (0.77–1.42)	0.17 (-0.09–0.43)
Region								
East Asia	12.22 (9.13–16.13)		1.02 (0.76–1.34)		8.22 (5.76–11.23)		1.11 (0.78–1.52)	-0.05 (-0.35–0.25)
Southeast Asia	6.58 (4.94–8.7)		1.09 (0.82–1.44)		6.42 (4.84–8.39)		1.23 (0.93–1.6)	0.54 (0.35–0.74)
Oceania	0.11 (0.08–0.14)		1.02 (0.74–1.35)		0.23 (0.17–0.31)		1.19 (0.86–1.56)	0.6 (0.43–0.76)
Central Asia	0.93 (0.69–1.22)		0.99 (0.74–1.3)		0.89 (0.67–1.2)		0.98 (0.74–1.32)	-0.05 (-0.17–0.07)
Central Europe	1.32 (0.99–1.69)		1.65 (1.23–2.1)		0.77 (0.55–1)		1.49 (1.07–1.94)	-0.33 (-0.44∼-0.22)
Eastern Europe	2.65 (1.95–3.48)		1.89 (1.39–2.48)		1.55 (1.15–2.05)		1.43 (1.06–1.89)	-0.36 (-0.51∼-0.21)
High-income Asia Pacific	1.92 (1.5–2.45)		2.03 (1.59–2.6)		1.32 (1.02–1.66)		2 (1.53–2.51)	0.28 (0.16–0.4)
Australasia	0.36 (0.29–0.45)		2.4 (1.9–2.96)		0.38 (0.28–0.5)		2.17 (1.56–2.85)	-0.54 (-0.66∼-0.42)
Western Europe	4.6 (3.78–5.53)		2.06 (1.7–2.48)		4.35 (3.45–5.38)		2.1 (1.67–2.6)	-0.16 (-0.33–0.01)
Southern Latin America	1.17 (0.92–1.51)		2.34 (1.84–3.01)		1.17 (0.93–1.47)		2.53 (2.02–3.17)	1.02 (0.74–1.3)
High-income North America	3.15 (2.34–4.07)		1.43 (1.06–1.85)		2.51 (1.98–3.04)		1.25 (0.98–1.51)	-1.06 (-1.31∼-0.81)
Caribbean	0.86 (0.64–1.14)		1.97 (1.48–2.63)		0.94 (0.7–1.29)		2.41 (1.78–3.3)	0.86 (0.7–1.02)
Andean Latin America	0.7 (0.54–0.9)		1.22 (0.94–1.56)		0.71 (0.55–0.91)		1.13 (0.88–1.44)	-0.37 (-0.48∼-0.25)
Central Latin America	3.87 (3.05–4.95)		1.63 (1.28–2.08)		3.22 (2.45–4.12)		1.53 (1.16–1.96)	0 (-0.14–0.15)
Tropical Latin America	3.69 (2.92–4.79)		2.16 (1.7–2.8)		3.01 (2.34–3.81)		1.95 (1.52–2.47)	-0.45 (-0.63∼-0.27)
North Africa and Middle East	12.09 (9.14–15.57)		2.16 (1.63–2.78)		12.95 (9.56–17.58)		2.23 (1.64–3.02)	0.09 (-0.09–0.26)
South Asia	13.19 (9.77–17.41)		0.77 (0.57–1.01)		12.7 (9.48–16.7)		0.79 (0.59–1.04)	0.37 (0.1–0.65)
Central Sub-Saharan Africa	1.51 (1.13–1.98)		1.18 (0.88–1.55)		2.39 (1.74–3.18)		1.12 (0.82–1.49)	-0.19 (-0.3∼-0.09)
Eastern Sub-Saharan Africa	4.21 (3.13–5.49)		0.99 (0.73–1.29)		6.22 (4.64–8.19)		0.92 (0.69–1.21)	-0.15 (-0.38–0.07)
Southern Sub-Saharan Africa	1.07 (0.79–1.4)		1.45 (1.07–1.9)		1.08 (0.82–1.41)		1.35 (1.03–1.77)	-0.22 (-0.36∼-0.07)
Western Sub-Saharan Africa	3.85 (2.88–5.03)		0.9 (0.67–1.18)		7.39 (5.49–9.74)		0.94 (0.7–1.24)	0.24 (0.02–0.46)

Note: ASIR, age-standardized incident rate per 100,000 population; EAPC, annual percentage change.

### Prevalence of Down Syndrome

The prevalent cases of all the SDI regions for both sexes were increasing except for the high-middle SDI region in the past three decades (Figure 1B and Table 2). The prevalent cases of DS reached 1,579,784 (95%UI 1,251,955-1,962,089) in 2019 from 1,257,110 (95%UI 989,416-1,573,671) in 1990. The ASPR of DS decreased from 1990 to 1997 and subsequently increased from 1998 to 2019 around the world (Table 2). At the regional level, the high SDI region had the most ASPR both in 1990 (44.67, 95%UI 36.06–54.88) and in 2019 (49.11, 95%UI 39.84–59.50), while low SDI region had the lowest in 1990 (11.83, 95% UI 8.96–15.42) and in 2019 (13.53, 95%UI 10.30–17.65) all the time. From the data in Figure 1B and Supplementary Figure S1B, males had more prevalent cases and ASPR than females regardless of the SDI region. From 1990 to 2019, prevalent cases between 15 and 49 years old were the largest group of DS patients in the global and in different SDI regions except low SDI region.

**TABLE 2:** Prevalence of Down syndrome in 1990/2019 and temporal trends.

Characteristic	1990				2019			1990–2019
	Prevalent case no.×10^3^ (95% CI)		ASPR/10^5^ no. (95% CI)		Prevalent cases no.×10^3^ (95% CI)		ASPR/10^5^ no. (95% CI)	EAPC no. (95% CI)
Overall	1257.11 (989.42–1573.67)		21.18 (16.71–26.54)		1579.78 (1251.95–1962.09)		21.51 (16.98–26.79)	0.09 (-0.04–0.21)
Sex								
Male	687.89 (539.06–860.68)		22.67 (17.82–28.42)		875.71 (694.18–1094.87)		23.37 (18.47–29.27)	0.12 (0.01–0.23)
Female	569.22 (449.47–713.06)		19.63 (15.5–24.53)		704.08 (558.97–870.37)		19.53 (15.48–24.24)	0.04 (-0.09–0.18)
Socio-demographic factor								
High SDI	332.64 (268.82–409.11)		44.67 (36.06–54.88)		384.95 (314.48–464.44)		49.11 (39.84–59.5)	0.08 (-0.03–0.19)
High-middle SDI	313.94 (248.45–392.42)		27.21 (21.55–34.04)		285.49 (226.45–353.59)		25.84 (20.37–32.09)	0.02 (-0.11–0.15)
Middle SDI	335.95 (258.08–429.31)		16.5 (12.67–21.14)		424.53 (330.33–535.98)		19.81 (15.43–25.1)	0.77 (0.62–0.92)
Low-middle SDI	185.72 (141.88–242.46)		12.75 (9.74–16.61)		280.46 (215.52–358.7)		14.86 (11.41–19)	0.66 (0.5–0.82)
Low SDI	88 (66.25–114.99)		11.83 (8.96–15.42)		203.05 (153.82–265.88)		13.53 (10.3–17.65)	0.55 (0.4–0.71)
Region								
East Asia	174.58 (130.1–225.49)		13.16 (9.82–17.02)		150.8 (114.99–197.29)		14.25 (10.79–18.62)	0.47 (0.31–0.63)
Southeast Asia	97.84 (74.63–125.41)		17 (13–21.71)		140.66 (109.39–178.07)		22.02 (17.07–27.94)	1.05 (0.78–1.32)
Oceania	1.04 (0.77–1.37)		12.25 (9.08–16.11)		2.41 (1.78–3.16)		14.91 (11–19.59)	0.66 (0.55–0.78)
Central Asia	14.62 (10.95–19.01)		17.74 (13.34–22.95)		18.31 (14–24.16)		19.21 (14.69–25.35)	0.36 (0.23–0.48)
Central Europe	29.28 (23.21–35.99)		26.9 (21.38–33.19)		18.6 (14.78–23.17)		24.11 (19.02–29.98)	0.25 (-0.3–0.8)
Eastern Europe	60.13 (46.96–76.15)		31.29 (24.27–39.58)		38.23 (29.48–48.38)		26.55 (20.36–33.92)	-0.21 (-0.34∼-0.08)
High-income Asia Pacific	76.14 (59.58–97.29)		50.13 (39.36–63.84)		71.38 (54.62–89.26)		60.05 (45.94–75.66)	0.8 (0.69–0.91)
Australasia	8.39 (6.63–10.49)		44.78 (35.42–55.77)		11.86 (9.32–14.94)		50.74 (39.96–64.22)	0.35 (0.3–0.39)
Western Europe	209.73 (175.02–249.84)		62.59 (52.22–74.62)		221.74 (183.68–267.97)		65.4 (54.03–79.11)	-0.01 (-0.15–0.13)
Southern Latin America	26.08 (20.19–33.38)		50.59 (39.23–64.8)		39.37 (30.25–49.64)		64.95 (49.88–81.99)	1.49 (1.2–1.78)
High-income North America	85.22 (64.52–112.35)		32.85 (24.87–43.19)		115.5 (92.37–138.71)		39.65 (31.67–47.64)	-0.15 (-0.41–0.11)
Caribbean	11.4 (9.2–14.31)		28.7 (23.2–35.75)		16.07 (12.83–20.24)		36.42 (28.91–46)	0.95 (0.87–1.04)
Andean Latin America	7.24 (5.48–9.44)		15 (11.41–19.58)		12.02 (9.09–15.39)		18.37 (13.89–23.53)	0.64 (0.56–0.72)
Central Latin America	60.44 (46.82–77.23)		28.96 (22.64–36.95)		63.88 (49.38–79.77)		25.99 (20.06–32.51)	-0.06 (-0.23–0.12)
Tropical Latin America	56.46 (44.1–71.67)		31.43 (24.61–39.73)		60.99 (46.66–77.83)		30.44 (23.32–39.01)	-0.46 (-0.71∼-0.22)
North Africa and Middle East	124.03 (94.23–159.62)		27.17 (20.59–34.87)		215.58 (162.68–278.82)		33.98 (25.71–44.06)	0.76 (0.64–0.87)
South Asia	139.62 (104.63–182.12)		9.93 (7.51–12.92)		218.32 (166.29–283.09)		11.49 (8.76–14.88)	0.7 (0.49–0.92)
Central Sub-Saharan Africa	8.84 (6.71–11.54)		11.14 (8.42–14.35)		21.6 (16.52–28.19)		12.19 (9.33–15.81)	0.34 (0.25–0.44)
Eastern Sub-Saharan Africa	26.57 (20.21–34.35)		9.63 (7.29–12.51)		57.96 (44.11–75.51)		10.38 (7.93–13.45)	0.35 (0.18–0.52)
Southern Sub-Saharan Africa	12.41 (9.35–16.22)		18.5 (13.93–24.09)		16.34 (12.52–21.14)		19.39 (14.88–25.1)	0.28 (0.08–0.48)
Western Sub-Saharan Africa	27.05 (20.59–34.87)		10.28 (7.91–13.27)		68.15 (52.55–87.82)		11.25 (8.7–14.43)	0.38 (0.26–0.49)

Note: ASPR, age-standardized prevalent rate per 100,000 population; EAPC, annual percentage change.

By contrasting the data in Table 1 and Table 2, there was greater inter-regional variation in prevalence compared to incidence. The EAPC of ASPR was positive in most regions apart from Central Europe, Eastern Europe, Western Europe, Central Latin America, High-income North America, and Tropical Latin America, which signified that the ASPR was on the rise in the past 30 years in most regions. From 1990 to 2019, Western Europe presented the highest prevalent cases and ASPR with no significant annual variability (Figure 3B and Table 2). Southern Latin America, which held the second place of ASPR (64.95, 95%UI 49.88–81.99) in 2019, had the highest EAPC of ASPR (1.49, 95%UI 1.20–1.78). Also, the region with the lowest EAPC of ASPR went to Tropical Latin America (-0.46, 95%UI −0.71to −0.22) (Figure 4B and Table 2). At the national level, Malta (98.86, 95%UI 79.10–122.11), Brunei Darussalam (97.20, 95%UI 74.28–122.16), and Ireland (93.00, 95%UI 73.15–116.70) were the top three countries of highest ASPR in 2019, with Brunei Darussalam and Ireland also having the highest ASIR (Figure 3A, Figure 3B and Supplementary Figure S3). The three nations with the fastest dropping ASPR were the same as the ASIR: Serbia (EAPC 2.09, 95%UI: −2.22 to −1.96), Bulgaria (EAPC -1.75, 95%UI -2.06 to-1.44), and Spain (EAPC -1.66, 95%UI −2.01 to −1.31). Argentina (1.96, 95% UI 1.65–2.28) and Georgia (1.75, 95% UI 1.46–2.03) had the highest EAPC of ASPR (Figure 4B and Supplementary Figure S4). We can find that the ASPR was positively correlated with SDI (R = 0.58, *p* < 0.0001) (Figure 5B) which corresponded to the data in Table 2.

### DALYs of Down Syndrome

The DALYs attributable to DS were on a downside globally from 2,223,900 (95%UI 1,499,510-4,609,110) in 1990 to 1,783,570 (95%UI 1,374,190-2,747,160) in 2019 (Table 3). In the globe, age-standardized DALYs declined by 25.95% gradually from 35.14 per 100,000 population (95%UI 23.81–72.35) in 1990 to 26.02 (95%UI 19.83–40.75) with an EAPC value of -1.05 (95% UI -1.11 to -0.99). Contrary to the global changing trend, the high SDI region and low SDI region had an uptrend in DALYs (Figure 1C). The middle SDI region got the highest number of DALYs both in 1990 and 2019 as the low SDI region had the lowest. The high SDI region retained the lowest of age-standardized DALYs in 1990 and 2019, while the highest age-standardized DALYs changed from the high-middle region to the low SDI region (Table3). The EAPC of age-standardized DALYs was negative except for the High SDI region in both sexes (Supplementary Figure S1C and Table 3).

**TABLE 3:** DALYs of Down syndrome in 1990/2019 and temporal trends.

Characteristic	1990				2019			1990-2019
	DALYs no.×10^3^ (95% CI)		Age-standardized DALYs/10^5^ no. (95% CI)		DALYs no.×10^3^ (95% CI)		Age-standardized DALYs/10^5^ no. (95% CI)	EAPC no. (95% CI)
Overall	2223.9 (1499.51–4609.11)		35.14 (23.81–72.35)		1783.57 (1374.19–2747.16)		26.02 (19.83–40.75)	-1.05 (-1.11∼-0.99)
Sex								
Male	1105.43 (794.99–3000.29)		33.92 (24.59–91.05)		906.95 (717.84–1565.92)		25.74 (20.22–45.11)	-1.02 (-1.1∼-0.93)
Female	1118.47 (572.5–2427.22)		36.43 (18.92–78.56)		876.62 (613.17–1444.44)		26.33 (18.11–44.13)	-1.09 (-1.13∼-1.04)
Socio-demographic factor								
High SDI	123.75 (109–145.2)		18.38 (16.22–21.58)		179.48 (146.1–201.2)		20.88 (17.27–23.75)	0.44 (0.31–0.56)
High-middle SDI	427.52 (347.12–608.37)		41.24 (33.42–59.17)		207.39 (181.33–239.95)		22.34 (19.33–26.28)	-2.57 (-2.78∼-2.36)
Middle SDI	781.08 (584.46–1338.65)		38.23 (28.68–65.31)		397.17 (337.48–468.63)		20.96 (17.75–24.94)	-2.27 (-2.42∼-2.13)
Low-middle SDI	495.16 (276.52–1236.09)		29.84 (17.08–72.96)		413.31 (306.24–605.06)		23.79 (17.59–34.96)	-0.64 (-0.69∼-0.59)
Low SDI	395.14 (110.43–1304.26)		41.19 (13.03–131.6)		584.64 (296.07–1334.3)		35.43 (18.96–78.49)	-0.29 (-0.46∼-0.12)
Region								
East Asia	615.17 (457.6–947.03)		50.72 (37.7–78.28)		133.57 (105.89–169.88)		16.05 (12.53–20.77)	-4.62 (-5.11∼-4.13)
Southeast Asia	84.8 (48.41–196.83)		14.55 (8.41–33.39)		89.49 (73.41–107.91)		15.54 (12.67–18.94)	0.33 (0.27–0.39)
Oceania	1.97 (0.75–5.67)		20.57 (8.25–57.65)		5.09 (2.18–12.99)		27.95 (12.46–69.94)	1.37 (1.17–1.57)
Central Asia	11.34 (8.57–19.67)		12.55 (9.56–21.42)		8.8 (6.58–11.11)		9.39 (7.01–11.87)	-1.48 (-1.88∼-1.08)
Central Europe	18.62 (15.56–27.26)		20.03(16.58–30.02)		9.43 (7.64–11.37)		12.55 (10.05–15.5)	-1.99 (-2.22∼-1.76)
Eastern Europe	46.2 (36.9–52.34)		28.2 (22.19–32.23)		20.05 (15.77–25.35)		14.82 (11.21–19.13)	-3.3 (-3.7∼-2.89)
High-income Asia Pacific	22.96 (19.1–27.01)		19.69 (16.51–23.09)		12.13 (9.52–15.22)		11.75 (9.22–14.52)	-1.46 (-1.87∼-1.05)
Australasia	3.25 (2.84–3.82)		18.55 (16.13–21.75)		6.13 (4.59–7.22)		24.76 (18.38–29.78)	0.83 (0.54–1.12)
Western Europe	66.67 (57.89–80.06)		22.4 (19.4–27.16)		95.87 (73.67–108.83)		24.94 (19.99–28.64)	0.67 (0.56–0.79)
Southern Latin America	21.6 (17.76–29.08)		42.74 (35.13–57.5)		24.54 (18.24–30.11)		46.29 (34.27–57.67)	0.66 (0.44–0.88)
High-income North America	32.84 (29–38.87)		13.13 (11.64–15.45)		67.48 (55.41–74.83)		19.12 (15.97–21.43)	1.1 (0.76–1.44)
Caribbean	18.02 (10.31–33.52)		45.33 (26.38–81.79)		21.22 (11.35–35.2)		49.55 (26.07–82.6)	0.43 (0.37–0.48)
Andean Latin America	20 (13.8–32.62)		36.24 (25.1–58.91)		19.46 (13.4–26.67)		30.55 (21.02–41.86)	-0.92 (-1.44∼-0.39)
Central Latin America	63.6 (55.92–78.59)		28.49 (25.16–34.95)		64.12 (46.04–82.79)		28.17 (20.16–36.52)	0.07 (-0.1–0.23)
Tropical Latin America	137.44 (71.91–217.08)		80.31 (42–126.71)		102.47 (80.64–143.93)		60.14 (46.55–85.79)	-1.24 (-1.38∼-1.09)
North Africa and Middle East	377 (218.23–872.73)		70.81 (41.35–161.96)		261.56 (204.88–354.15)		43.5 (34–58.89)	-1.62 (-1.69∼-1.56)
South Asia	305.52 (134.62–834.08)		19.91 (9.4–52.17)		244.47 (153.69–349.63)		14.47 (8.99–20.91)	-0.99 (-1.08∼-0.9)
Central Sub-Saharan Africa	47.62 (12.66–163.07)		44.39 (13.4–146.7)		60.07 (31.63–122.27)		31.2 (17.04–61.73)	-1.12 (-1.22∼-1.02)
Eastern Sub-Saharan Africa	141.59 (34.48–447.2)		39.26 (10.82–120.04)		197.01 (102.09–406.42)		31.79 (17.16–64.79)	-0.42 (-0.7∼-0.13)
Southern Sub-Saharan Africa	22.15 (16.43–30.7)		31.8 (23.86–43.72)		27.67 (21.15–35.61)		34.53 (26.36–44.44)	0.36 (0.1–0.62)
Western Sub-Saharan Africa	165.55 (36.55–575.78)		46.4 (11.76–159.48)		312.95 (115.38–848.89)		44.01 (17.28–115.77)	0.03 (-0.06–0.13)

Note: EAPC, annual percentage change.

Regionally, the DALYs were found to be the highest in East Asia in 1990 (615,170, 95%UI 457,600-947,030) and in Western Sub-Saharan Africa in 2019 (312,950, 95%UI 115,380-848,890) (Table3). Tropical Latin America was the top one in terms of the age-standardized DALYs both in 1990 (80.31, 95%UI 42.00–126.71) and in 2019 (60.14, 95%UI 46.55–85.79). On the contrary Central Asia held the lowest age-standardized DALYs in 1990 (12.55, 95%UI 9.56–21.42) and in 2019 (9.39, 95%UI 7.01–11.87) (Table 3). East Asia had the most rapid decline of age-standardized DALYs in the past three decades with an EAPC value of -4.77 (95%UI −5.11 to −4.13) (Table 3 and Figure 4C).

At the country/territory level, Paraguay (82.26, 95%UI 59.98–122.98), Haiti (80.39, 95%UI 22.55–165.20), and Algeria (79.28, 95%UI 49.15–128.46) had the highest age-standardized DALYs in 2019. Palau (4.56, 95%UI 3.19–6.34), Cook Islands (4.91, 95%UI 2.63–8.64), as well as Romania (5.35, 95%UI 4.15–6.88) had the lowest one (Figure 3C and Supplementary Table S3). Among the countries mentioned previously, Haiti and Palau also had relatively high and low ASIRs, respectively, as previously written. Guatemala (7.14, 95%UI 5.72–8.57), Georgia (4.72, 95%UI 3.88–5.56), and Bahrain (4.37, 95%UI 3.58–5.18) had the highest EAPC of age-standardized DALYs. Serbia (−7.48, 95%UI −8.31 to -6.64), China (−4.71, 95%UI −5.22 to −4.20), and Uzbekistan (−4.67, 95%UI −5.52 to −3.81) had the lowest EAPC of age-standardized DALYs (Figure 4C and Supplementary Table S4). The SDI negatively correlated with age-standardized DALYs (R = 0.37, *p* < 0.0001), which meant that the more developed region may be more likely to have lower DALYs (Figure 5C).

### Death of Down Syndrome

At the global level, the number of deaths due to DS had a slide downward trend in the last 30 years from 28,380 cases (95%UI 16,900-52,830) in 1990 to 22,280 (95% UI 17,760-33,170) in 2019 (Table 4). The age-standardized death rate (ASDR) and their changing trends varied among different SDI regions and countries. Interestingly, the distribution and variation related to death were similar to those in DALYs (Figures 3C,D, Figures 4C,D, Table 3 and Table 4). The high-middle SDI region held the highest ASDR (0.46, 95%UI 0.37–0.67) in 1990, while the low SDI region (0.43, 95%UI 0.24–0.93) had the highest in 2019 (Table 4). Same as DALYs, the ASDR was gradually declining in all except the high SDI region (Supplementary Figure S1D, Table 4). Most of the patients who died were younger than 5 years old in all SDI regions except high SDI, in which the majority of deaths were among patients over 50 years old.

**TABLE 4:** Death of Down syndrome in 1990/2019 and temporal trends.

Characteristic	1990				2019			1990–2019
	Death no.×10^3^ (95% CI)		ASDR/10^5^ no. (95% CI)		Death no.×10^3^ (95% CI)		ASDR/10^5^ no. (95% CI)	EAPC no. (95% CI)
Overall	25.38 (16.9–52.83)		0.41 (0.27–0.84)		22.28 (17.76–33.17)		0.32 (0.25–0.48)	-0.84 (-0.89∼-0.79)
Sex								
Male	12.51 (9–34.33)		0.39 (0.28–1.05)		11.26 (9.14–18.82)		0.31 (0.25–0.53)	-0.77 (-0.84∼-0.7)
Female	12.87 (6.54–28.19)		0.42 (0.22–0.92)		11.03 (7.94–17.61)		0.32 (0.22–0.53)	-0.9 (-0.95∼-0.85)
Socio-demographic factor								
High SDI	1.47 (1.35–1.77)		0.21 (0.19–0.25)		3.29 (2.47–3.6)		0.3 (0.24–0.33)	1.49 (1.3–1.68)
High-middle SDI	4.8 (3.87–6.9)		0.46 (0.37–0.67)		2.61 (2.28–3)		0.26 (0.22–0.3)	-2.49 (-2.7∼-2.28)
Middle SDI	8.82 (6.56–15.25)		0.44 (0.33–0.75)		4.63 (3.91–5.49)		0.24 (0.2–0.29)	-2.27 (-2.42∼-2.13)
Low-middle SDI	5.7 (3.18–14.24)		0.36 (0.21–0.86)		4.93 (3.63–7.16)		0.29 (0.21–0.41)	-0.62 (-0.67∼-0.58)
Low SDI	4.58 (1.31–15.02)		0.5 (0.17–1.56)		6.81 (3.49–15.44)		0.43 (0.24–0.93)	-0.3 (-0.46∼-0.15)
Region								
East Asia	6.92 (5.11–10.65)		0.57 (0.42–0.88)		1.47 (1.16–1.89)		0.17 (0.13–0.23)	-4.77 (-5.27∼-4.26)
Southeast Asia	0.92 (0.51–2.22)		0.16 (0.09–0.38)		0.99 (0.8–1.22)		0.17 (0.14–0.21)	0.28 (0.19–0.36)
Oceania	0.02 (0.01–0.07)		0.25 (0.1–0.69)		0.06 (0.02–0.15)		0.33 (0.15–0.83)	1.36 (1.16–1.56)
Central Asia	0.12 (0.09–0.22)		0.13 (0.1–0.24)		0.09 (0.07–0.12)		0.1 (0.07–0.13)	-1.61 (-2.05∼-1.17)
Central Europe	0.21 (0.18–0.31)		0.22 (0.18–0.33)		0.13 (0.1–0.16)		0.15 (0.12–0.19)	-1.74 (-1.97∼-1.51)
Eastern Europe	0.51 (0.41–0.58)		0.31 (0.24–0.36)		0.24 (0.19–0.31)		0.16 (0.12–0.22)	-3.46 (-3.91∼-3.01)
High-income Asia Pacific	0.2 (0.17–0.23)		0.18 (0.15–0.21)		0.09 (0.08–0.11)		0.09 (0.07–0.1)	-2.32 (-2.93∼-1.7)
Australasia	0.04 (0.04–0.05)		0.23 (0.2–0.28)		0.11 (0.08–0.12)		0.35 (0.25–0.42)	1.24 (0.92–1.57)
Western Europe	0.81 (0.73–1.03)		0.25 (0.22–0.32)		1.8 (1.23–1.99)		0.36 (0.26–0.4)	1.9 (1.7–2.11)
Southern Latin America	0.25 (0.2–0.34)		0.49 (0.41–0.67)		0.3 (0.22–0.37)		0.54 (0.4–0.67)	0.72 (0.51–0.92)
High-income North America	0.43 (0.4–0.53)		0.16 (0.15–0.2)		1.41 (1.09–1.53)		0.32 (0.25–0.35)	2.27 (1.86–2.69)
Caribbean	0.23 (0.13–0.41)		0.61 (0.37–1.03)		0.3 (0.17–0.51)		0.68 (0.36–1.14)	0.48 (0.43–0.53)
Andean Latin America	0.23 (0.16–0.37)		0.41 (0.29–0.68)		0.22 (0.15–0.3)		0.35 (0.23–0.48)	-0.89 (-1.42∼-0.35)
Central Latin America	0.7 (0.61–0.87)		0.32 (0.28–0.4)		0.79 (0.57–1.02)		0.34 (0.24–0.44)	0.32 (0.14–0.49)
Tropical Latin America	1.55 (0.8–2.46)		0.92 (0.48–1.45)		1.28 (1.03–1.8)		0.72 (0.57–1.03)	-1.01 (-1.15∼-0.87)
North Africa and Middle East	4.26 (2.43–9.96)		0.81 (0.47–1.87)		2.97 (2.3–4.11)		0.49 (0.38–0.69)	-1.66 (-1.73∼-1.6)
South Asia	3.62 (1.63–9.75)		0.25 (0.13–0.63)		3.05 (1.93–4.35)		0.18 (0.11–0.26)	-1.03 (-1.1∼-0.96)
Central Sub-Saharan Africa	0.55 (0.15–1.88)		0.54 (0.18–1.73)		0.71 (0.37–1.44)		0.39 (0.22–0.77)	-1.03 (-1.13∼-0.94)
Eastern Sub-Saharan Africa	1.63 (0.4–5.15)		0.48 (0.14–1.41)		2.29 (1.19–4.76)		0.39 (0.22–0.77)	-0.42 (-0.7∼-0.15)
Southern Sub-Saharan Africa	0.25 (0.19–0.35)		0.38 (0.29–0.52)		0.33 (0.25–0.42)		0.41 (0.32–0.53)	0.34 (0.1–0.58)
Western Sub-Saharan Africa	1.92 (0.43–6.67)		0.56 (0.16–1.87)		3.64 (1.34–9.76)		0.53 (0.22–1.35)	0.04 (-0.05–0.13)

Note: ASIR, age-standardized incident rate per 100,000 population; ASDR, age-standardized death rate per 100,000 population; EAPC, annual percentage change.

At the regional scale, East Asia had the largest number of deaths in 1990 (6,920, 95%UI 5,110-10,650) with the lowest EAPC of ASDR (−4.77, 95%UI -5.27 to −4.26) (Table 2). Central Asia displayed the lowest and the second-lowest ASDR in 1990 (0.13, 95%UI 0.10–0.24) and 2019 (0.10, 95%UI 0.07–0.13), respectively. Tropical Latin America had the highest ASDR both in 1990 (0.92, 95%UI 0.48–1.45) and 2019 (0.72, 95%UI 0.57–1.03) (Figure 3D, Figure 4D and Table 4).

The ASDR was the highest in Haiti (1.20, 95%UI 0.30–2.66), followed by Paraguay (1.00, 95%UI 0.65–1.48) and Algeria (0.91, 95%UI 0.56–1.53) in 2019, which were also top 3 countries in the ranking of the age-standardized DALYs in 2019. The three countries with the lowest ASDR in 2019 were San Marino (0.04, 95%UI 0.02–0.06), Romania (0.05, 95%UI 0.03–0.06), and Palau (0.05, 95%UI 0.03–0.07) (Figure 3D and Supplementary Table S3).

As reported in Figure 4D and Supplementary Table S4, the fastest growth of ASDR was in Guatemala (EAPC 7.95, 95% UI 6.35–9.56), the United Kingdom (EAPC 6.34, 95%UI 5.29–7.40), and Georgia (EAPC 5.22, 95%UI 4.31–6.14), whereas the fastest decrease was in Serbia (EAPC −7.44, 95%UI −8.28 to −6.60), Uzbekistan (EAPC −5.38, 95%UI −6.34 to −4.40), and Singapore (EAPC −5.19, 95%UI −6.31 to −4.06). Importantly, Georgia was in the top three in all the EAPC rankings while Serbia was consistently in the top three fastest declining of those (Figures 4A–D and Supplementary Table S4). The ASDR was negatively correlated with SDI (R = −0.36, *p* < 0.0001), which meant that the more developed region might be more likely to have lower ASDR (Figure 5).

## Discussion

DS is one of the main causes of intellectual disability, and these patients face a variety of health problems (Asim et al., 2015). Charleton et al. once formulated almost 44 particular medical problems that existed more regularly in people with DS (Stores and Stores, 2013). DS imposes a huge medical and social cost; therefore, an exploration of the global burden and trends of DS through SDI and region stratification is a valuable reference for public health leaders, researchers, and clinical doctors. In this study, we noticed that the incident cases, ASIR, and ASPR declined slightly first, then increased, especially in recent 5 years, and ultimately were unchanged in the past three decades. The prevalent cases were rising around the world, whereas the number and ASRs of DALYs and deaths decreased gradually from 1990 to 2019. Substantial diversity of disease burden and trends were discovered in different SDI or geographic regions, and individual countries. In addition, the ASIR and ASPR had a positive correlation with corresponding SDI value; on the contrary, age-standardized DALYs and ASDR had a negative correlation with SDI.

The results from early studies demonstrated that human aneuploidy may be induced by environmental factors, such as chemotherapy, cigarette smoking, endocrine-disrupting chemicals, or exogenous hormones (Munne et al., 1997; Yang et al., 1999; Frias et al., 2003; Allard and Colaiacovo, 2010; Brieno-Enriquez et al., 2011). It is challenging to identify such factors due to multifactorial nature of the process and the potential complexity of the interactions.

The increasingly widespread practice of prenatal screening and selective termination of pregnancy have exerted significant positive impact on alleviating the burden of DS. de Graaf et al. (2015) estimated an overall 30% reduction in the numbers of babies with DS from 2006 to 2010 due to elective pregnancy terminations. Although gynecologists and midwives are legally obliged to inform each pregnant woman about the options for prenatal screening at the booking visit, the willingness to receive those tests is not the same, despite the absolute benefit of prenatal screening. Morris showed that receipt of antenatal diagnosis was observed in 70% of mothers aged greater than 37 years, while in 43% of younger mothers in England and Wales (Morris and Alberman, 2009). Many expectant couples choose not to pursue prenatal screening or diagnostic test altogether (de Graaf et al., 2017). Two studies reported that antenatal diagnosis occurred in about 15% of mothers in Arab Emirates and Ireland, which were relatively high-income countries (Corder et al., 2017). There is no doubt that this reason partially explained why Ireland was in the top three countries with the highest number and ASRs of incident and prevalent cases. Researchers also found the lower participation rates in prenatal test among women from a lower socioeconomic background (Kuppermann et al., 2006; Rowe et al., 2008; Fransen et al., 2010). The percentage of women aged greater than 35, who do not have universal screening, prenatal diagnosis, and associated services, was high in middle- and low-income countries (Christianson et al., 2006). Keeping in line with that finding, we observed the incident cases in high SDI region showed a downward trend but in the low SDI region presented an upward trend. In addition, an important finding was reported that, especially women from Turkish and Moroccan ethnic origin were less likely to participate in prenatal screening for DS in Dutch even after adjustment for differences in socioeconomic background and age (Fransen et al., 2010). The white race was much more aware of the availability terminating fetuses, and therefore, the impact of this procedure was greater among the white race than among those of other races after identification with DS (Krivchenia et al., 1993). These differences in prenatal screening and termination could partly be attributed to policies, provisions, and uptake of prenatal screening, socioeconomic background, awareness, and ethnic and religious beliefs**.** Those differences between countries partially lead to wide variation in incidence and prevalence of DS. Earlier detection of DS prenatally and selective termination of pregnancy timely could undoubtedly decrease burden of DS. Therefore, it would be meaningful to take the prenatal screening and selective termination of pregnancy to equip healthcare programs in less developed countries.

It is well known that advancing maternal age increases the risk of DS (Loane et al., 2013). In the 1980s, women started waiting to have children until later years (de Graaf et al., 2017). The mean age of women at the birth of their first child increased across all European Union Member States in the last three decades (EuropenState, 2015).‘Late childbirth’ is an increasingly popular trend in many countries and regions (Kenny et al., 2013). Technically, advanced maternal age refers to women who are 35 years of age or older at the time of the delivery of her baby. Loane et al. (2013) found that ten out of twelve European countries reported more than 50% of mothers to be 35 years of age or older). In our study, relatively stable number and ASRs of incident were observed in different regions and in the global level except for the low SDI region from 1990 to 2019. This is consistent with other studies reporting no increasing trends in many areas of the world (Baird and Sadovnick, 1988; Loane et al., 2013). The increasingly widespread termination of pregnancy had, on average, counteracted the effect of maternal age and resulted in a relatively stable incidence of DS in different regions except for the low SDI region. This overall change of incident cases globally may be attributed to a decline in the number of incident cases in high, high-middle, and middle SDI, offset by an increase in the low SDI region. The increased and upward trends of incidence in low SDI region should raise concern, which poses a serious challenge to children health.

The most common cause of death in childhood and adulthood with DS remains respiratory infection, while congenital heart defects cause most deaths in early childhood (Bull, 2020). Children with DS were now mandated by federal law to have their congenital heart defects repaired, leading to another boost in childhood survival rates (de Graaf et al., 2017). Experience from high-income countries shows that up to 70% of birth defects can either be prevented, or that affected children can be offered care, which could be lifesaving or would reduce the severity of disability. These interventions include appropriate treatment, particularly surgery and prevention, especially before conception or in very early pregnancy (Christianson et al., 2006). Better medication and improved care for complications related to DS have increased the life expectancy (Verstegen and Kusters, 2020). In our study, high SDI region showed the less incident cases of DS than lower SDI region but higher ASIR in the past three decades when the impacts of ageing were removed by converting counts number to ASRs. In the meantime, high SDI region suffered from higher number and ASRs of prevalent cases than those of low SDI region. Moreover, the SDI was positively correlated with the ASIR and ASPR in 21 regions from 1990 to 2019. Based on the definition of SDI and the aforementioned finding, we suggested that the incidence and prevalence of patients with DS might be associated with SDI level, medical service condition, and community support.

The higher incidence of birth with DS has also been reported in consanguineous marriage (CM) families, which suggests families with consanguinity might be a risk factors for DS (Corder et al., 2017)^,^ (Ray et al., 2018). Consanguinity results in an excess of homozygosity for recessive traits and has been reported in association with a higher frequency of cardiac malformations in patient with DS (El Mouzan et al., 2008). CM is a common practice in many parts of the world, especially in the Eastern Mediterranean and North Africa (Christianson et al., 2006). Regional differences are distinctive in our study. We found the relative higher number and ASRs of incident and prevalent cases in North Africa and Middle East partially attributable to common CM in those regions.

In line with a previous article, there were differences in the burden of DS according to the geographic region (Bull, 2020). Geographical variation was great in incidence and prevalence of DS in our study. We found that Ireland was among the top three countries with the highest ASIR and ASPR. Termination of pregnancy for fetal anomaly was illegal in Ireland and Malta. Therefore, it was potentially owing to differences in maternal age and prenatal screening (Nordstrøm et al., 2020); in addition to that, willingness to participate in prenatal screening, healthcare policy, and national legislation should also be taken into account (Churchill et al., 2012). Furthermore, Western Europe remained the first place of prevalent cases and ASPR all the time; we also found that the ASPR was positively correlated with SDI, which meant that ASPR seemed to be higher in relatively developed regions. To our knowledge, in developed countries, relatively good success had been made to meet the needs of children with DS through an early intervention procedure. As a result, they lived longer and healthier in the community in a family environment due to life-saving advances in health care, such as pediatric cardiac surgery and modified social housing (Churchill et al., 2012). Interestingly, Georgia is in the top three in all the EAPC rankings, while Serbia is consistently in the top three fastest declining of those. We should pay more attention to these two countries to get a better understanding of promoting or inhabiting factors influencing DS burden and trends.

It has been previously described that the proportion of males is higher in patients with DS. (Takeuchi et al., 2008; Glivetic et al., 2015). Consistent with previous studies, our study found that the males prevalent cases exceeded that of females regardless of the SDI region, which might be related to a differential rate for fetus survival *in utero* between sexes (Bishop et al., 1997; Agopian et al., 2012; Corona-Rivera et al., 2019).

The DALYs and death toll increased in both high and low SDI over the past three decades. After eliminating the influence of different age compositions, the trend of age-standardized DALY rate and ASDR in those regions were distinctive with an upward trend in high SDI region and a downward trend in low SDI region. Christianson et al. (2006) reported that 65% of the infants and children with DS had died by the age of two in South Africa in the early and mid-1990s. Coinciding with the report in Brazil that children were more susceptible to death (de Campos Gomes et al., 2020), the majority of deaths occurred in the children less than 5 years old in different SDI region except high SDI region. Prioritizing a reduction in deaths of newborns and children is one of the seventeen Sustainable Development Goals (Nations, 2014). To achieve this goal to fall well below United Nations projections, more efforts should be made to decrease the death of DS globally. In addition, we found that most of the deaths were over 50 years old in high SDI region. This might be due to high proportion of older DS patients in high SDI region where DS patients have a longer life expectancy. Both age-standardized DALYs and ASDR were negatively correlated with SDI, indicating that difference might be partly attributed to social and economic factors. Analogously, many studies indicated that sociodemographic characteristics impacted the survival and the risk of mortality for patients with DS (Fiscella et al., 2000; de Campos Gomes et al., 2020). According to the literature, the most common reason of death in childhood and adulthood remained as respiratory infection (Bull, 2020); dementia was the direct reason of death in 70% of dead older people with DS (McGlinchey et al., 2020). Cardiovascular and pulmonary diseases accounted for ∼75% of the death rate in people with DS (Colvin and Yeager, 2017). Without doubt, improved medical care for complications related to DS has increased the life expectancy of DS and decreased mortality and morbidity in good economic-social countries and regions (Verstegen and Kusters, 2020). The lives of persons with DS and of their families had been improved greatly by guidelines development, their propagated extension to medical staff, and advancement in medical treatment protocol with social support (Crissman et al., 2006; Asim et al., 2015). The United States reported a remarkable 46% decline in infant mortality rates from birth defects over the period 1980 to 2001, and much of this reduction can be attributed to improvements in diagnosis, care, and prevention (Christianson et al., 2006).

Our research is the first comprehensive report on the DS epidemiology, which fills a gap in the global burden and trends of DS. People with DS are living longer than they have before. Increased utilization of prenatal testing and DS-related elective terminations had counterbalancing effects on population growth, resulting in relatively stable numbers of people with DS (de Graaf et al., 2017). However, several limitations should be noted when interpreting our results. First, the diagnosis of DS might be underreported and may result in bias in DS registration, especially in nations with limited medical resources or low economic regions, which could lead to an underestimation of the disease burden. Second, as data are lacking in some parts of the world, information bias is unavoidable with respect to the epidemiologic assessment of DS. Third, the roles of risk factors (except SDI) for DS were not estimated in this study. Some risk factors might help to explain geographic and temporal patterns in the disease burden.

In general, our results implied that substantial diversities of DS burden and trends were across different regions and countries with different sociodemographic characteristics. Our results implied that significant improvement had been made in reducing DALYs and deaths worldwide from 1990 to 2019. However, the increased number and ASRs of incident and prevalent cases in some regions, especially in low SDI regions, are raising concerns, which pose a serious challenge to children health. We hope to draw the attention of policy makers in order to facilitate funding and resources allocation. Information provided by this article should help to elucidate the global disease burden and trends of DS and to build more effective intervention.

## Data Availability

The datasets used in the present study are available in the Global Burden of Disease 2019.

## Abbreviations

ASR, age-standardized rate; ASIR, age-standardized incident rate; ASDR, age-standardized death rate; CI, certainty interval; DALY, disability-adjusted life year; DS, Down syndrome; EAPC, estimated annual percentage change; GBD, global burden of disease; SDI, social-demographic index.
