# Supplementary material for: Global, Regional, and National Burden and Trends of Down Syndrome From 1990 to 2019
Source: Front Genet. 2022 Jul 15;13:908482. doi: 10.3389/fgene.2022.908482 (PMC9337874; doi:10.3389/fgene.2022.908482)
Supplement: Supplementary file 7 [file DataSheet1.docx]

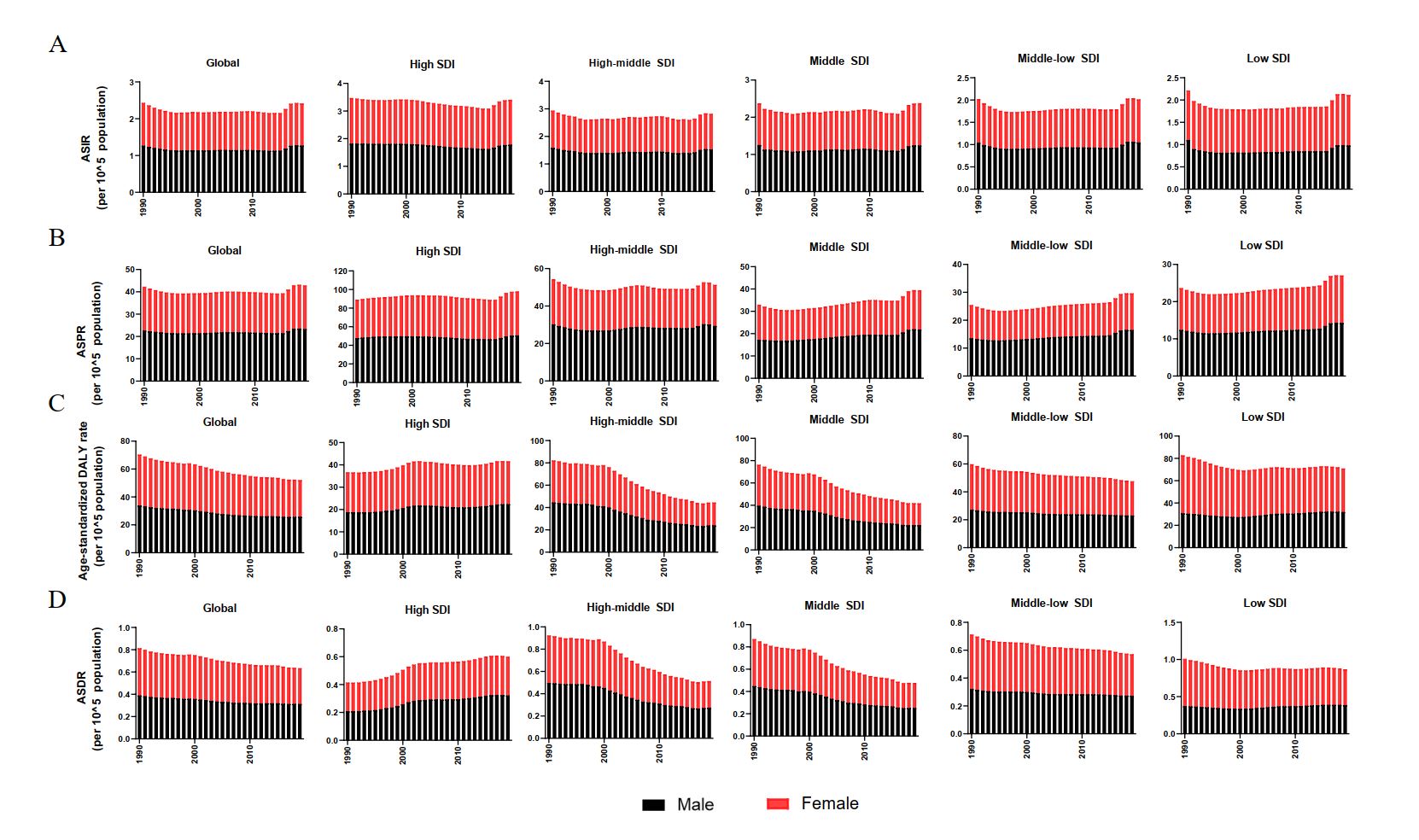


Figure S1. The burden and trends of DS globally and in five SDI quintiles from 1990 to 2019. (A) Age-standardized incident rate (ASIR). (B) Age-standardized prevalent rate (ASPR). (C) Age-standardized DALY rates. (D) Age-standardized death rates (ASDR). Note: DALYs, disability-adjusted life-years. SDI, social-demographic index.
